# The association of individual cognition and social environment of smoking with autonomy over tobacco: A survey from rural China

**DOI:** 10.18332/tid/175974

**Published:** 2024-01-19

**Authors:** Jiaoyan Li, Yimei Zhu, Zhihong Zhang, Deyu Cai, Huinan Han, Jing Liang, Fang Wang, Beizhu Ye, Yuan Liang

**Affiliations:** 1 Department of Social Medicine and Health Management, School of Public Health, Tongji Medical College, Huazhong University of Science and Technology, Wuhan, China; 2 School of Media, Communication and Sociology, University of Leicester, Leicester, United Kingdom; 3 Department of Oncology, Gongan County People's Hospital, Hubei Province, China; 4 Department of Academic Research, Hubei Academy of Social Sciences, Wuhan, China; 5 Department of Epidemiology and Biostatistics, College of Public Health, Xuzhou Medical University, Xuzhou, China; 6 Department of Social Medicine and Health Management, College of Public Health, Zhengzhou University, Zhengzhou, China

**Keywords:** individual cognition, social environment, the autonomy over tobacco, smoke

## Abstract

**INTRODUCTION:**

This study explores the association of individual cognition and social environment of smoking with autonomy over tobacco, providing evidence and insights to help smokers effectively prevent and reduce tobacco dependence.

**METHODS:**

Data were collected from 1389 participants, aged ≥15 years, by face-to-face interviews from June 2018 to November 2019 in central China. We assessed autonomy over tobacco using the Autonomy Over Smoking Scale (AUTOS), including Withdrawal Symptoms (WS), Psychological Dependence (PD) and Cue-induced Cravings (CC), and examined factors of individual cognition and social environment, as well as covariates, including demographic characteristics, health status, and smoking behavior.

**RESULTS:**

AUTOS total score was 16.92 ± 9.05, WS score was the lowest (4.40 ± 3.36) in the three subscales, and CC score was the highest (6.88 ± 3.2). After adjustment, WS score of having a greater awareness of smoking hazards to one's own health was lower than those who had no awareness (β=0.14; 95% CI: -0.31–0.00), and the total score of AUTOS, the score of PD and CC for those who thought smoking was ‘more helpful (high)’ to interpersonal communication were higher than ‘not helpful (not at all)’ (β=0.14; 95% CI: 0.01–0.28 with β=0.16; 95% CI: 0.02–0.29; and β=0.14; 95% CI: 0.00–0.28; respectively). Having a greater difficulty in smoking cessation was associated with higher AUTOS total and subscale scores (p<0.001). Notably, none of the social-environmental factors included had a significant association with AUTOS scores.

**CONCLUSIONS:**

Interventions targeting individual cognitive factors of tobacco dependence seem to be more effective in smoking cessation. Future research may explore the influence of family and workplace among social environmental factors, which may reveal the effect of a binding force.

## INTRODUCTION

Tobacco dependence has been observed among people, similar to many other drugs^1^. Tobacco smoking is considered an unhealthy behavior, contributing to the increased risk of developing chronic non-communicable diseases (NCDs), whilst tobacco use disorder (usually referred to as tobacco dependence) has been defined as a mental illness^2^, which itself is an NCD. Compared to the usual tobacco smoking, although all tobacco use can be considered problematic since there is no safe level of use, tobacco use disorder would reveal the differences in the development stages of tobacco use, as well as the corresponding intervention strategies.

Despite the well-documented dangers of tobacco use and a majority desire for smoking cessation, the long-term cessation success rate remains near 4% among tobacco users^3^. There are two possible sequencing stages of the process: tobacco use prevention, which is more important, and treating tobacco use disorder as an NCD. In the two stages of this process, the external social environment is more closely associated with tobacco use, and intrinsic individual cognition may have more influence on tobacco use disorder^4^. Some studies have shown that smokers who are more dependent on tobacco tend to respond more to internal and external cues^5^. Therefore, understanding the characteristics of intrinsic cognition and its correlation with tobacco use disorder may be the starting point or premise for an in-depth analysis of the complexity of smoking and smoking cessation interventions.

Compared with studies on the influencing factors of tobacco smoking, studies on the influencing factors of tobacco use disorder are relatively few. At present, the influencing factors of tobacco dependence mostly focus on sociodemographics, smoking characteristics^6-8^, and psychological distress^9^. Some studies have also explored the clinical characteristics of patients with specific diseases^10-14^. Although some studies have analyzed the impact of beliefs about smoking and environmental factors on loss of autonomy^15,16^, they mainly focused on adolescents and targeted waterpipe use, and there is little in-depth analysis of individual cognitive and social environmental factors for tobacco use disorder. In addition, no prior studies have explored the factors or initiatives aimed at promoting autonomy over tobacco in China^2,17^.

In China, the average prevalence of tobacco use was 26.6% in 2018, significantly higher in men than in women (50.5% vs 2.1%); the smoking rate in rural areas (28.9%) was higher than that in urban areas (25.1%)^17^, and the average prevalence of tobacco use disorder was 13.1%. Among smokers, the prevalence of tobacco use disorder was 49.7%, with no difference between men and women (49.7% vs 50.8%)^2^.

We conducted a cross-sectional survey study in rural China to investigate the degree of autonomy over tobacco among smokers, and to explore the association of individual cognition and the social environment of smoking with autonomy over tobacco. The findings will provide evidence and insights to help smokers effectively prevent and reduce tobacco dependence.

## METHODS

### Design and sample

Data collection and training of the investigators who collected survey data, door-to-door and face-to-face, were carried out from June 2018 to November 2019 in rural areas in Hubei Province, located in central China. The research team obtained ethics approval from the review board of Tongji Medical College, Huazhong University of Science and Technology [2019-S006]. Two counties were selected from Hubei Province by purposive sampling, and convenience sampling was used to select 4–5 villages in each of the two counties. All households of the selected villages were included and were visited by at least one of the investigators. The final analytic study sample was 1389 participants, aged >15 years (mean=59.9, SD=15.0), including 254 smokers (defined as smoking cigarettes or tobacco other than cigarettes in the past 30 days), and without hearing or speech impairment, mental or other serious illness.

### Measures

The explanatory variables included aspects of individual cognition and social environment. Three individual cognition factors were assessed with four questions from three aspects: 1) Harms of smoking^7,15^, ‘How do you evaluate the hazards of smoking on the health of that smoker?’ and ‘How do you evaluate the hazards of smoking on the health of others?’; 2) Benefits of smoking^15^, ‘How do you evaluate the benefits of smoking on interpersonal communication?’; and 3) Perception of one’s own ability to stop smoking (the self-efficacy of quitting smoking)^18^, ‘How do you evaluate the degree of difficulty in stop smoking?’. The responses to these four questions were coded as: 0= ‘Not at all’, 1= ‘Low’, and 2= ‘High’. Social environment factors included three aspects: health services utilization, family environment, and workplace environment. Health services utilization was assessed with the question, ‘How often do your doctors advise you to quit smoking?’ and was recorded as low or high. Family^15^ and workplace factors were measured with three questions each, respectively: ‘Does your home have non-smoking regulations?’, ‘Do your direct relatives smoke?’, ‘Do your elders smoke^19^?’, ‘Does your workplace have non-smoking regulations^20^?’, ‘Do your colleagues smoke?’, and ‘Do your leaders smoke?’. The answers were recoded as binary variables: no (0) vs yes (1).

Outcome variables were assessed using the 12-item Autonomy Over Smoking Scale (AUTOS) (range: 0–36) ^21^ since AUTOS offers more insights from three symptoms, namely Withdrawal Symptoms (WS), Psychological Dependence (PD), and Cue-induced Cravings (CC) (range: 0–12). Individuals with higher scores experienced less autonomy. The internal consistency, as shown by Cronbach’s alpha of the overall AUTOS, was high (α=0.92). Cronbach’s alpha for the sub-scales of WS, PD, and CC were 0.83, 0.76, and 0.79.

We included control variables to address potential confounding by demographic characteristics (gender, age, education level, marital status, self-reported economic status), health-related characteristics (body mass index, number of outpatient visits and hospitalizations in the last year) and smoking behaviors (age at smoking onset, time to the first cigarette of the day), and number of cigarettes smoked per day)^15^.

### Statistical analysis

We first examined the distribution of smoking behavior and its relationship with the characteristics and health status. The outcome variables included total scores for AUTOS and scores for each of the three symptoms.

To determine which factors influenced the use of tobacco, we performed a logistic regression analysis among the total sample. Independent-sample t-tests and the test for trend, which was performed with a polynomial contrast procedure, were used to test AUTOS scores as continuous variables of smokers. We also compared group differences of critical factors using the Mann-Whitney U test/Kruskal-Wallis test. All explanatory variables significant at p<0.05 in the bivariate models were entered into linear regression models.

Furthermore, we conducted several sensitivity analyses exploring the integration of social and environmental factors. Family environment consisted of smoking status of direct relatives and elders (range: 0–2), work environment consisted of smoking status of colleagues or leaders (range: 0–2), no-smoking rules at home or workplace (range: 0–2), and all 7 factors as an exposure factor (range: 0–7) with ridge regression analysis; including only male respondents; only adult respondents aged <85 years; and classified analysis of outcome variable scores by quartile. Statistical significance was evaluated with 2-sided tests, with the level of significance at p<0.05. All analyses were conducted in IBM SPSS Statistics, Version 26.0 (IBM Corp, Armonk, NY).

## RESULTS

Demographic characteristics of the studied total sample by tobacco use status are presented in Table 1. Females account for 59.6% of the sample. The smoking rate was 18.29% (95% CI: 16.26–20.32), whilst men were 28 times (AOR=28.1; 95% CI: 17.1–46.2) more likely to smoke than women. In particular, being overweight (AOR=0.68; 95% CI: 0.47–0.98) and having been hospitalized within a year (AOR=0.67; 95% CI: 0.46–0.99) were protective factors for smoking behavior.

**Table 1:** Demographic characteristics of the studied total sample, by tobacco use status, in central China, 2018–2019 (N=1389)

*Characteristics*	*Overall* *%*	*Tobacco use*		*OR (95%CI)*	*AOR (95%CI)^a^*
		*Yes* *%*	*No* *%*		
**Total sample**		18.29	81.71		
**Gender**					
Female ®	59.61	7.87	71.19	1	1
Male	40.39	92.13	28.81	28.91 (17.99–46.46)***	28.10 (17.10–46.18)***
**Age** (years)					
≤49 ®	19.29	13.39	20.62	1	1
50–59	23.33	17.32	24.67	1.08 (0.67–1.75)	1.2 (0.68–2.10)
60–69	29.52	36.61	27.93	2.02 (1.32–3.10)**	1.82 (1.07–3.09)**
≥70	27.86	32.68	26.78	1.88 (1.22–2.90)**	1.53 (0.88–2.65)
**Education level**					
Illiterate ®	28.80	18.11	31.19	1	1
Primary school	35.49	39.37	34.63	1.96 (1.34–2.86)***	0.96 (0.60–1.54)
Junior high school	27.00	30.71	26.17	2.02 (1.36–3.00)***	1.00 (0.60–1.68)
Senior high school and higher	8.71	11.81	8.02	2.54 (1.52–4.24)***	0.97 (0.50–1.86)
**Marital status**					
Married ®	82.29	81.10	82.56	1	1
Single/divorced/widowed/other	17.71	18.90	17.44	1.10 (0.78–1.56)	1.04 (0.69–1.58)
**Economic status**					
Low	55.51	49.61	56.83	0.74 (0.56–0.98)	0.79 (0.57–1.11)
Normal ®	39.60	45.28	38.33	1	1
Well-off	4.90	5.12	4.85	0.89 (0.47–1.69)	0.64 (0.31–1.34)
**Health status**					
**BMI** (kg/m^2^)					
≤18.4	9.22	12.20	8.55	1.18 (0.76–1.84)	1.29 (0.76–2.20)
18.5–23.9 ®	54.42	63.39	52.51	1	1
≥24	36.23	24.41	38.94	0.52 (0.38–0.71)	0.68 (0.47–0.98)**
**Number of outpatient visits in the last year**					
0–2 ®	39.60	71.65	65.73	1	1
≥3	33.19	28.35	34.27	0.76 (0.56–1.02)	0.86 (0.60–1.24)
**Number of hospitalizations in the last year**					
0 ®	71.85	74.80	71.19	1	1
≥1	28.15	25.20	28.81	0.83 (0.61–1.14)	0.67 (0.46–0.99)**

a AOR: adjusted odds ratio; adjusted for gender, age, education level, marital and economic status.

® Reference categories. BMI: body mass index.

** p<0.05.

*** p<0.001.

Table 2 shows demographic characteristics, health status, smoking behavior, individual cognition and social environment of the smoker sample by autonomy over tobacco. The mean AUTOS total score was 16.92 (SD=9.05; range: 0–36). The sub-scale score of WS (4.40 ± 3.36; range: 0–12) was the lowest in the three subscales, and the CC score (6.88 ± 3.2; range: 0–12) was the highest. The results of the test for trend shows that a lower level of education was associated with a higher score of WS (p=0.021), suggesting that smokers with lower educational level experienced more severe withdrawal symptoms. Approximately 57% of smokers in this study believed that smoking had no harm or low harm (lower risk perception) to themselves. In total, 40% of smokers admitted that tobacco played an important role in social interaction, and 70% reported that quitting smoking was extremely difficult. The test for trend indicated that smokers who believed in the benefit of smoking for interpersonal communication and those with lower scores on the smoking cessation self-efficacy assessment had higher scores of AUTOS (p<0.05). About 70% of smokers reported that their direct relatives or elders smoked, while only 16.54% and 15.75% of them had non-smoking regulations at home or workplace, respectively. Another 53.94% of smokers reported that they were advised to quit smoking frequently by doctors.

**Table 2:** Demographic characteristics, health status, smoking behavior, individual cognition and social environment of tobacco user sample, by autonomy over tobacco, in central China, 2018–2019 (N=254)

*Characteristics*	*Overall* *%*	*The autonomy over smoking scale*							
		*Total*		*Withdrawal symptoms*		*Psychological dependence*		*Cue-induced cravings*	
		*Mean ± SD*	*p*	*Mean ± SD*	*p*	*Mean ± SD*	*p*	*Mean ± SD*	*p*
**Total**		16.92 ± 9.05		4.40 ± 3.36		5.64 ± 3.25		6.88 ± 3.25	
**Gender^a^**									
Female	7.87	14.60 ± 11.36	0.233	4.45 ± 4.17	0.947	4.60 ± 3.93	0.136	5.55 ± 3.85	0.057
Male	92.13	17.12 ± 8.83		4.40 ± 3.29		5.73 ± 3.18		6.99 ± 3.18	
**Age^b^** (years)									
≤49	13.39	15.97 ± 8.38	0.589	4.40 ± 3.64	0.546	5.68 ± 3.17	0.995	6.26 ± 2.91	0.382
50–59	17.32	16.41 ± 9.51		4.56 ± 3.23		5.27 ± 3.37		6.77 ± 3.51	
60–69	36.61	17.86 ± 8.35		4.36 ± 3.34		5.98 ± 3.06		7.32 ± 2.95	
≥70	32.68	16.53 ± 9.85		4.03 ± 3.12		5.45 ± 3.45		6.69 ± 3.54	
**Education level^b^**									
Illiterate	18.11	16.37 ± 9.93	0.069	4.46 ± 3.84	0.021	5.37 ± 3.43	0.309	6.54 ± 3.46	0.095
Primary school	39.37	17.96 ± 8.87		4.75 ± 3.21		5.88 ± 3.25		7.33 ± 3.21	
Junior high school	30.71	17.59 ± 9.16		4.59 ± 3.49		5.91 ± 3.33		7.09 ± 3.16	
Senior high school and higher	11.81	12.57 ± 6.72		2.67 ± 2.07		4.57 ± 2.65		5.33 ± 2.93	
**Marital status^a^**									
Married	81.10	17.03 ± 9.23	0.695	4.46 ± 3.42	0.591	5.65 ± 3.28	0.969	6.93 ± 3.28	0.618
Single/divorced/widowed/other	18.90	16.46 ± 8.33		4.17 ± 3.09		5.63 ± 3.17		6.67 ± 3.16	
**Economic status^b^**									
Low	49.61	18.19 ± 9.13	0.941	4.93 ± 3.36	0.941	6.02 ± 3.29	0.826	7.24 ± 3.18	0.929
Normal	45.28	15.37 ± 8.82		3.76 ± 3.16		5.16 ± 3.23		6.45 ± 3.33	
Well-off	5.12	18.38 ± 8.75		5.00 ± 4.20		6.23 ± 2.77		7.15 ± 3.11	
**Health status**									
**BMI** (kg/m^2^)^b^									
≤18.4	12.20	16.84 ± 9.10	0.651	4.32 ± 3.16	0.601	5.84 ± 3.33	0.545	6.68 ± 3.20	0.911
18.5–23.9	63.39	17.32 ± 9.34		4.60 ± 3.48		5.70 ± 3.32		7.02 ± 3.25	
≥24	24.41	15.94 ± 8.29		3.94 ± 3.14		5.40 ± 3.07		6.6 ± 3.31	
**Number of outpatient visits in the last year**									
0–2	71.65	16.64 ± 9.80	0.755	4.17 ± 3.58	0.484	5.60 ± 3.39	0.891	6.88 ± 3.47	0.993
≥3	28.35	17.03 ± 8.76		4.49 ± 3.27		5.66 ± 3.21		6.88 ± 3.17	
**Number of hospitalizations in the last year**									
0	74.80	16.72 ± 9.09	0.836	4.45 ± 3.2	0.887	5.61 ± 3.22	0.927	6.66 ± 3.37	0.529
≥1	25.20	16.99 ± 9.06		4.38 ± 3.42		5.65 ± 3.28		6.95 ± 3.22	
**Smoking behavior**									
**Age at smoking onset^a^** (years)									
<18	16.54	18.26 ± 9.31	0.294	4.93 ± 3.64	0.266	5.83 ± 3.43	0.677	7.50 ± 3.11	0.175
≥18	83.46	16.66 ± 9.00		4.30 ± 3.30		5.60 ± 3.23		6.75 ± 3.27	
**Time to the first cigarette of the day**									
≥60	30.31	13 ± 8.64	<0.001	3.04 ± 3.05	<0.001	4.26 ± 2.95	<0.001	5.7 ± 3.26	<0.001
31–59	10.63	16.33 ± 8.07		4.37 ± 3.13		5.33 ± 2.99		6.63 ± 2.95	
6–30	22.83	16.21 ± 7.74		4.05 ± 2.83		5.38 ± 2.90		6.78 ± 2.82	
≤5	36.22	20.83 ± 8.97		5.77 ± 3.49		7.05 ± 3.27		8.00 ± 3.25	
**Number of cigarettes smoked per day^b^**									
≤10	27.56	11.6 ± 8.20	<0.001	2.87 ± 2.88	<0.001	3.7 ± 2.94	<0.001	5.03 ± 2.95	<0.001
11–20	46.46	18.37 ± 8.39		4.81 ± 3.21		6.3 ± 2.97		7.27 ± 3.18	
21–30	12.20	19.97 ± 9.08		5.45 ± 3.52		6.29 ± 3.52		8.23 ± 2.94	
≥31	13.78	19.97 ± 8.62		5.17 ± 3.67		6.74 ± 3.03		8.06 ± 2.81	
**Individual cognition**									
**Smoking hazards to one’s own health^b^**									
Not at all	30.71	18.87 ± 9.11	0.235	5.38 ± 3.39	0.026	6.31 ± 3.3	0.276	7.18 ± 3.21	0.932
Low	26.38	14.03 ± 7.86		3.43 ± 2.95		4.63 ± 2.83		5.97 ± 2.93	
High	42.91	17.30 ± 9.33		4.29 ± 3.41		5.79 ± 3.34		7.22 ± 3.39	
**Smoking hazards to others’ health^b^**									
Not at all	29.92	16.5 ± 8.77	0.597	4.36 ± 3.28	0.949	5.46 ± 3.22	0.543	6.68 ± 3.04	0.425
Low	26.38	16.91 ± 9.77		4.48 ± 3.41		5.66 ± 3.44		6.78 ± 3.66	
High	43.70	17.22 ± 8.86		4.39 ± 3.41		5.76 ± 3.18		7.07 ± 3.15	
**Smoking benefits to interpersonal communication^b^**									
Not at all	31.89	15.53 ± 9.35	0.001	4.20 ± 3.31	0.037	5.04 ± 3.46	<0.001	6.30 ± 3.33	<0.001
Low	28.35	14.15 ± 8.14		3.47 ± 3.01		4.75 ± 2.94		5.93 ± 3.00	
High	39.76	20.01 ± 8.58		5.23 ± 3.46		6.76 ± 2.99		8.02 ± 3.04	
**Difficulty in smoking cessation^b^**									
Not at all	12.20	7.61 ± 6.53	<0.001	1.58 ± 2.14	<0.001	2.26 ± 2.34	<0.001	3.77 ± 3.01	<0.001
Low	14.17	10.14 ± 6.87		2.00 ± 2.45		3.42 ± 2.26		4.72 ± 2.92	
High	73.62	19.77 ± 8.01		5.33 ± 3.19		6.63 ± 2.95		7.81 ± 2.82	
**Social environment**									
**Doctor’s advice^b^**									
Not at all	19.69	16.52 ± 9.43	0.267	4.28 ± 3.20	0.255	5.52 ± 3.58	0.433	6.72 ± 3.41	0.260
Low	26.38	14.67 ± 8.66		3.46 ± 3.09		5.12 ± 3.27		6.09 ± 3.18	
High	53.94	18.17 ± 8.93		4.91 ± 3.46		5.94 ± 3.11		7.32 ± 3.17	
**No smoking regulations at home^a^**									
No	83.46	16.86 ± 9.12	0.819	4.46 ± 3.39	0.518	5.63 ± 3.29	0.875	6.77 ± 3.25	0.251
Yes	16.54	17.21 ± 8.78		4.10 ± 3.21		5.71 ± 3.09		7.40 ± 3.24	
**Direct relatives smoking^a^**									
No	24.80	15.19 ± 9.16	0.08	3.84 ± 3.17	0.127	5.11 ± 3.28	0.136	6.24 ± 3.25	0.072
Yes	75.20	17.49 ± 8.96		4.59 ± 3.41		5.82 ± 3.23		7.09 ± 3.23	
**Elders smoking^a^**									
No	33.46	15.86 ± 8.88	0.185	4.09 ± 3.15	0.301	5.27 ± 3.22	0.198	6.49 ± 3.25	0.183
Yes	66.54	17.46 ± 9.11		4.56 ± 3.45		5.83 ± 3.26		7.07 ± 3.24	
**No smoking regulations at workplace^a^**									
No	62.99	17.54 ± 9.3	0.004	4.61 ± 3.46	0.001	5.84 ± 3.35	0.056	7.08 ± 3.29	0.024
Yes	15.75	13.85 ± 6.27		3.08 ± 2.21		4.75 ± 2.62		6.03 ± 2.38	
**Colleagues smoking^a^**									
No	36.61	16.32 ± 9.73	0.425	4.40 ± 3.62	0.904	5.30 ± 3.45	0.190	6.62 ± 3.35	0.297
Yes	36.22	17.40 ± 8.60		4.34 ± 3.23		5.95 ± 3.21		7.12 ± 3.09	
**Leaders smoking^a^**									
No	40.16	16.26 ± 9.65	0.317	4.45 ± 3.65	0.671	5.25 ± 3.38	0.082	6.56 ± 3.36	0.131
Yes	30.71	17.65 ± 8.57		4.23 ± 3.15		6.13 ± 3.24		7.29 ± 3.03	

a Independent-sample t-tests between groups.

b Test for trend between groups, which was performed with a polynomial contrast procedure.

BMI: body mass index.

Supplementary file Tables S1 and S2 show the distribution of three critical individual cognition and social environment factors by demographic variables. The cognition of smoking hazards to one’s own health was significantly associated with age and economic status (p<0.05), indicating that older and richer smokers were less convinced of the harms of smoking to their health. The cognition of smoking benefits to interpersonal communication was significantly associated with gender and marital status, indicating that male and married smokers believed that smoking is more conducive to interpersonal communication. No significant association was found between smoking cessation self-efficacy/key social environment factors and demographic characteristics, except economic status and smoking cessation advice from doctors.

Table 3 shows the results of significant factors associated with adjusted autonomy over tobacco. WS score of having a high awareness of smoking hazards to health was on average 0.15 points lower than those who had no awareness (95% CI: -0.31–0.00), and the total score of AUTOS (adjusted β=0.14; 95% CI: 0.01–0.28), PD score (adjusted β=0.16; 95% CI: 0.02–0.29), and CC score (adjusted β=0.14; 95% CI: 0.00–0.28) for those who thought smoking was ‘more helpful (high)’ to interpersonal communication was higher than ‘not helpful (not at all)’. Having a greater difficulty in smoking cessation was associated with higher AUTOS total and subscale scores (p<0.001). Notably, none of the social-environmental factors had a significant association with AUTOS scores. In the results of sensitivity analyses, significant changes were not observed. Although we classified the social environmental factors, the results were very similar to those of the main analysis (Supplementary file Tables S3, S4 and S5).

**Table 3:** Linear regression analysis of significant factors on autonomy over tobacco, in central China, 2018–2019 (N=254)

	*Autonomy over smoking scale*							
	*Total*		*Withdrawal symptoms*		*Psychological dependence*		*Cue-induced cravings*	
	*Adjusted β^a^ (95% CI)*	*p*	*Adjusted β^a^ (95% CI)*	*p*	*Adjusted β^a^ (95% CI)*	*p*	*Adjusted β^a^ (95 % CI)*	*p*
**Individual cognition**								
**Smoking hazards to one’s own health** (Ref: Not at all)								
Low	-0.09 (-0.23–0.06)	0.234	-0.13 (-0.28–0.03)	0.110	-0.11 (-0.25–0.04)	0.145	0 (-0.15–0.15)	0.973
High	-0.09 (-0.23–0.06)	0.227	-0.15 (-0.31–0.00)	0.049	-0.09 (-0.24–0.05)	0.200	0.01 (-0.14–0.16)	0.887
**Smoking benefits to interpersonal communication** (Ref: Not at all)								
Low	-0.03 (-0.17–0.11)	0.669	-0.05 (-0.19–0.1)	0.545	0 (-0.14–0.14)	0.975	-0.04 (-0.18–0.11)	0.596
High	0.14 (0.01–0.28)	0.039	0.09 (-0.05–0.23)	0.223	0.16 (0.02–0.29)	0.022	0.14 (0–0.28)	0.048
**Difficulty in smoking cessation** (Ref: Not at all)								
Low	0.12 (-0.04–0.28)	0.132	0.1 (-0.06–0.27)	0.222	0.15 (-0.01–0.31)	0.062	0.07 (-0.09–0.24)	0.374
High	0.45 (0.29–0.61)	<0.001	0.39 (0.22–0.57)	<0.001	0.47 (0.31–0.63)	<0.001	0.38 (0.21–0.55)	<0.001
**Social environment**								
**No smoking regulations at the workplace** (Ref: No)								
Yes	-0.08 (-0.2–0.04)	0.195	-0.11 (-0.23–0.02)	0.101	-0.06 (-0.18–0.06)	0.361	-0.05 (-0.18–0.07)	0.411
**Covariate**								
**Education status** (Ref: Illiterate)								
Primary school	0.12 (-0.03–0.28)	0.124	0.08 (-0.10–0.25)	0.385	0.14 (-0.03–0.30)	0.098	0.13 (-0.03–0.29)	0.117
Junior high school	0.04 (-0.11–0.19)	0.595	0.02 (-0.14–0.19)	0.806	0.06 (-0.10–0.22)	0.442	0.03 (-0.13–0.19)	0.686
Senior high school and higher	-0.05 (-0.18–0.09)	0.485	-0.07 (-0.22–0.07)	0.321	0.01 (-0.13–0.15)	0.936	-0.06 (-0.21–0.08)	0.375
**Time to the first cigarette of the day** (Ref: ≥60 minutes)								
31–59	0.04 (-0.08–0.17)	0.499	0.1 (-0.03–0.24)	0.141	0.04 (-0.09–0.17)	0.548	-0.02 (-0.16–0.11)	0.717
6–30	0.1 (-0.03–0.24)	0.136	0.14 (-0.01–0.29)	0.068	0.1 (-0.04–0.24)	0.168	0.05 (-0.09–0.19)	0.496
≤5	0.22 (0.08–0.37)	0.002	0.22 (0.06–0.37)	0.006	0.24 (0.09–0.38)	0.002	0.16 (0.01–0.31)	0.037
**Number of cigarettes smoked per day** (Ref: ≤10)								
11–20	0.19 (0.04–0.33)	0.012	0.15 (-0.01–0.3)	0.061	0.19 (0.05–0.34)	0.010	0.17 (0.02–0.32)	0.028
21–30	0.13 (-0.01–0.26)	0.066	0.08 (-0.07–0.22)	0.288	0.09 (-0.04–0.23)	0.179	0.18 (0.04–0.32)	0.014
≥31	0.13 (-0.01–0.27)	0.070	0.05 (-0.1–0.2)	0.518	0.13 (-0.01–0.27)	0.076	0.18 (0.03–0.32)	0.016
**Adjusted R^2^**	0.368		0.271		0.356		0.303	

a Adjusted for educational level and smoking history and habits.

Further sensitivity analyses show that a similar distribution was observed for participants, including only males or only adults aged <85 (Supplementary file Tables S6 and S7). In addition, after adjustment for identical potential confounders and dividing the outcome variables by quartiles, almost only individual cognition factors were still significant between the total and three sub-scales scores (Supplementary file Tables S8 and S9).

## DISCUSSION

To our knowledge, this is the first study that provides empirical evidence to indicate an association of individual cognition and social environment of smoking with tobacco dependence. Overall, there were statistically significant correlations between individual cognitive factors and tobacco dependence, but not social environmental factors.

Participants from rural China were smokers at a rate of 18.29% (95% CI: 16.26–20.32), lower than the 26.6% reported in the China Adult Tobacco Survey 2018^17^. This is because almost 60% of the participants were female in this study, whilst a nationwide survey would have a higher rate of male participants who are, in general, more likely to smoke than women. The findings of this study showed that people who were overweight/obese or had been hospitalized in the previous year were less likely to smoke, indicating that poor health and serious illness can be protective factors for smoking behavior, as previous studies also found that the main reasons for smoking cessation are disease and disease prevention^22^.

Due to different sample sizes and gender ratios, on the whole, our sample was relatively close to the level of autonomy over tobacco reported by previous studies, although there were some differences. Our AUTOS score was 16.92 ± 9.05 (range: 0–36), and the sub-scale score of WS was 4.40 ± 3.36 (range: 0–12). A Hong Kong-based study with a baseline survey of smokers before smoking cessation therapy, showed that the AUTOS score was 19.33 ± 7.99 (range: 0–36), while the sub-scale score of WS was higher (6.33 ± 3.20, range: 0–12) ^23^.

In terms of individual cognition factors, smokers who believed in the benefit of smoking for interpersonal communication had less autonomy over tobacco, indicating that these smokers considered smoking as a social tool to build stronger connections, whilst this practice could potentially increase the frequency of smoking and the risk of dependence^24^. Over half of the smokers in this study had a lower risk perception of smoking, which is in line with previous studies, as smokers tended to underestimate the long- and short-term risks of tobacco consumption^4^. A previous study also showed that neither current smokers nor people with high tobacco dependence believed that light smoking carries any risk of lung cancer^25^. Moreover, there is a negative association between an individual’s perception of the health hazards of smoking and withdrawal symptoms, which is consistent with findings from previous studies that smokers with more pronounced withdrawal symptoms and stronger nicotine dependence held a lower risk perception of smoking^26^. Withdrawal symptom is a critical factor in maintaining smoking behavior^27^, in connection with a reduced likelihood of quitting^28^. Therefore, smokers with lower risk perception are less likely to quit smoking. Driven by anticipated feelings caused by previous experience and consolidated beliefs^29^, smokers’ perception of the risks of smoking will guide them to adopt protective behaviors. Therefore, when smokers learn more about the harm of smoking to their health, they will be more likely to quit.

Two previous studies conducted in India and Lebanon found that smokers’ perceptions of ‘whether health is harmful to individuals’^7,15^ and ‘whether smoking promotes interpersonal communication’^15^ were not associated with loss of autonomy, similar to what we found. It indicated that such cognition needed to reach a certain degree in order to effectively avoid and reduce the degree of tobacco dependence. In contrast, a study in France showed that smokers who were highly dependent on tobacco tended to be more aware of the risk of lung cancer caused by smoking than those who were not dependent^25^. Health literacy and cultural factors may play a role in such differences between Eastern and Western studies. In the future, not only more guidance and education are needed to make smokers fully aware of the harms of smoking, but longitudinal cohort studies on the causal relationship between risk perception and tobacco dependence are needed to fundamentally mitigate and reduce the degree of tobacco dependence of smokers.

Smoking cessation self-efficacy was positively associated with autonomy over tobacco, as smokers with lower levels of self-efficacy reported poorer levels of smoking cessation autonomy and higher levels of nicotine dependence. Reduced self-efficacy is related to smoking impulse, which may be linked to tobacco addiction. A previous study found that individuals with lower post-quit abstinence self-efficacy and greater depressive symptoms were less likely to recover from a lapse^30^. Self-efficacy determines the degree of effort that patients exert in the face of difficulties and setbacks^31^. When a smoker has high self-efficacy with a positive psychological state, he or she will put more effort into quitting smoking and accordingly reduce the degree of tobacco dependence. Good risk perception and self-efficacy may be effective factors for smokers to improve autonomy and reduce tobacco dependence^32^. Therefore, more recommendations and interventions are needed to improve the risk perception and self-efficacy of smokers and improve the individual cognition of smokers.

In terms of social and environmental factors, although some studies have found that doctors’ advice^18^, smokers in one’s family^19^ or in the workplace^20^ had a significant impact on tobacco use, these factors had no significant association with tobacco use disorder in this study. Social and environmental factors influencing tobacco use can be different from factors influencing tobacco use disorder. Hence, different prevention and control strategies should be adopted at different stages of smoking.

We found that smokers with non-smoking regulations at work had a higher degree of autonomy, whilst non-smoking regulations at home had no significant effect on autonomy. A study conducted in Europe on the impact of smoking bans had similar results that smoking bans were effective in reducing passive smoking in the workplace but not in residential smoking^33^. This current study was carried out in rural China, where people live in small, closed communities, and the smoking ban at home had little effect on autonomy. Workplace non-smoking regulations, on the other hand, are protected by law and are more binding. Tobacco bans not only protect non-smokers from passive exposure but also reduce smoking rates and cigarette consumption^34^. Consequently, when non-smoking regulations are implemented in the workplace, they will improve smokers’ autonomy to a certain extent due to their binding effect.

In this study, the association between social environment factors and AUTOS score was not significant after adjusting for demographic characteristics and individual cognition factors, but individual cognition factors still affected AUTOS score. This finding is consistent with a previous study on Chinese smokers that both prohibition norms and smoking risk perception were the influencing factors of smoking cessation intention, but the prediction of full mediation of smoking risk perception affected the smoking cessation intention of prohibition norms^35^. In other words, prohibition norms influence smoking cessation behavior through the full mediating effect of smoking risk perception. A study conducted in the United States also found similar findings that stricter residential smoking rules were associated with higher self-efficacy in treating children to reduce their tobacco smoke exposure^36^. This showed that although many studies described cue-induced cravings in autonomy as being influenced by social factors, or even though smoking temptation is more common in the social environment, a person’s own state will affect the effect of temptation on him. When an individual has sufficient personal cognition, the cue-induced craving for autonomy is not necessarily sufficient to resist the restraint consciousness of personal cognition for healthy behavior to avoid the smoking impulse of smokers.

### Limitations

Our study still has several limitations. First, there are still limitations of single indicators in the measurement of exposure factors, and more comprehensive factors need to be considered in the future. Second, the cross-sectional nature of this study precludes the ability to draw causal inferences and requires the compilation of longitudinal monitoring data on the autonomy over tobacco. Third, the study relies on self-report and is therefore susceptible to recall bias. Future research may explore the influence of family and workplace among social environmental factors, which may reveal the effect of the binding force. Fourth, the sample came from a province in a rural area of central China, which would limit generalizability to other areas and countries.

## CONCLUSIONS

This study analyzed the association of individual cognition and the social environment of smoking with autonomy over tobacco in a rural Chinese sample. The autonomy was negatively related to the effect of smoking on interpersonal communication and positively related to self-efficacy of smoking cessation. Moreover, individual perception of the health hazards of smoking also had a significant negative association with withdrawal symptoms. Therefore, interventions targeting individual cognitive factors of tobacco dependence may be more effective in smoking cessation.

## Data Availability

The data supporting this research are available from the authors on reasonable request.
